# Interactions between Rhodamine Dyes and Model Membrane Systems—Insights from Molecular Dynamics Simulations

**DOI:** 10.3390/molecules27041420

**Published:** 2022-02-19

**Authors:** Nisa Magalhães, Guilherme M. Simões, Cristiana Ramos, Jaime Samelo, Alexandre C. Oliveira, Hugo A. L. Filipe, João P. Prates Ramalho, Maria João Moreno, Luís M. S. Loura

**Affiliations:** 1 Coimbra Chemistry Center—Institute of Molecular Sciences (CQC-IMS), University of Coimbra, 3004-535 Coimbra, Portugal; nisa-tamara@hotmail.com (N.M.); guisims8@gmail.com (G.M.S.); cristianavramos95@gmail.com (C.R.); jsamelo@uc.pt (J.S.); ac_oliveira10@hotmail.com (A.C.O.); hlfilipe@ipg.pt (H.A.L.F.); mmoreno@ci.uc.pt (M.J.M.); 2 Department of Chemistry, Faculty of Sciences and Technology, University of Coimbra, 3004-535 Coimbra, Portugal; 3 CPIRN-IPG—Center of Potential and Innovation of Natural Resources, Polytechnic Institute of Guarda, 6300-559 Guarda, Portugal; 4 Hercules Laboratory, LAQV, REQUIMTE, Department of Chemistry, School of Science and Technology, University of Évora, 7000-671 Evora, Portugal; jpcar@uevora.pt; 5 CNC—Center for Neuroscience and Cell Biology, 3004-504 Coimbra, Portugal; 6 Faculty of Pharmacy, University of Coimbra, 3000-548 Coimbra, Portugal

**Keywords:** fluorescence, lipid bilayer, membrane probes, molecular dynamics, rhodamine, xanthene dyes

## Abstract

Background: rhodamines are dyes widely used as fluorescent tags in cell imaging, probing of mitochondrial membrane potential, and as P-glycoprotein model substrates. In all these applications, detailed understanding of the interaction between rhodamines and biomembranes is fundamental. Methods: we combined atomistic molecular dynamics (MD) simulations and fluorescence spectroscopy to characterize the interaction between rhodamines 123 and B (Rh123 and RhB, respectively) and POPC bilayers. Results: while the xanthene moiety orients roughly parallel to the membrane plane in unrestrained MD simulations, variations on the relative position of the benzoic ring (below the xanthene for Rh123, above it for RhB) were observed, and related to the structure of the two dyes and their interactions with water and lipids. Subtle distinctions were found among different ionization forms of the probes. Experimentally, RhB displayed a lipid/water partition coefficient more than two orders of magnitude higher than Rh123, in agreement with free energy profiles obtained from umbrella sampling MD. Conclusions: this work provided detailed insights on the similarities and differences in the behavior of bilayer-inserted Rh123 and RhB, related to the structure of the probes. The much higher affinity of RhB for the membranes increases the local concentration and explains its higher apparent affinity for P-glycoprotein reconstituted in model membranes.

## 1. Introduction

Rhodamine dyes are notable for their high absorption coefficient and intense fluorescence in the visible region of electromagnetic spectrum, as well as for their photostability [1]. For this reason, they are widely used as fluorescent probes. In this context, an increasingly large number of applications of rhodamines concerns the study of biological systems, including membranes and membrane proteins [2].

Biological membranes constitute natural obstacles to the absorption of drugs and their distribution in the body to reach the target tissue. The characterization of the interaction with and permeation through biological membranes for a large set of drug-like molecules is therefore of high importance, to gain predictive value in the development of drugs with improved bioavailability. Most xenobiotics permeate membranes by passive mechanisms [3], but their effective permeation is often limited by efflux proteins that transport drugs from the membrane to the aqueous media outside the cells. Among the efflux transporters, P-glycoprotein (P-gp) has been extensively studied, and its structure, mechanism, and wide range of substrates and inhibitors are well characterized [4,5,6,7,8,9,10,11,12]. Due to the excellent photophysical properties of rhodamines and the large number of derivatives available with a wide range of properties, rhodamines have contributed very significantly to the elucidation of P-gp specificity and mechanism [9,12].

In this work, we study the interaction of two rhodamine dyes, rhodamine 123 (Rh123) and rhodamine B (RhB), with membrane model systems. Rh123 is considered a model compound for the interaction with P-gp. At least two binding sites have been identified in P-gp, one of which has been named as “site R” due to interaction with rhodamine 123 [9]. Additionally, Rh123 has also been used as a sensitive probe of mitochondrial membrane potential [13,14,15].

**Figure 1:**
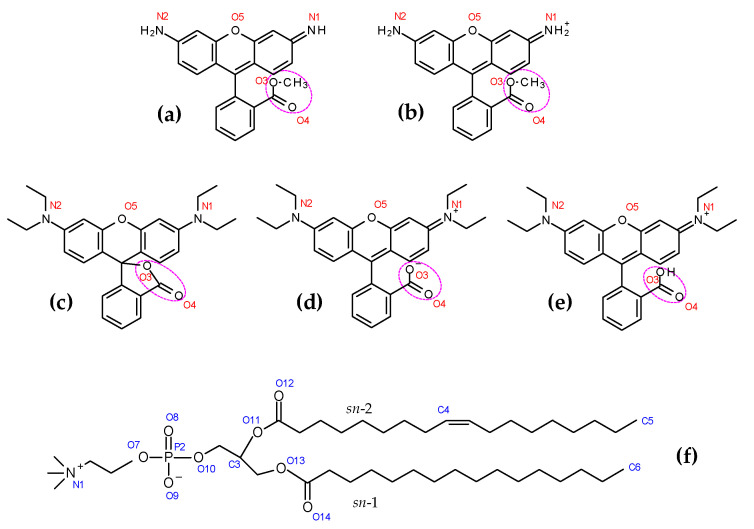
Structures of Rh123 (0) (**a**), Rh123 (+1) (**b**), RhB (lactone) (**c**), RhB (Zwitterion) (**d**), RhB (+1) (**e**) and POPC (**f**), with numbering of relevant atoms as mentioned in this article. The pink dotted ellipse in the rhodamine dyes indicates the ester (Rh123) or COO (RhB) group, as used below.

RhB is an inexpensive dye, commonly employed as fluorescent tag of proteins and lipid membrane probes. The free dye is structurally closely related to Rh123, differing solely on the substituents of the N atoms of the xanthenic ring system (which are H atoms in Rh123 and ethyl groups in RhB) and on the methyl esterification of the benzoic carboxylic moiety in Rh123 (see structures in Figure 1). Despite these seemingly small changes, RhB has been described as a poor P-gp substrate when compared with Rh123 [16]. While the reasons behind this different behavior are not entirely understood, the membrane interactions of the two probes, such as partition and rate of permeation, possibly represent key factors. The permeability of RhB, determined by the parallel artificial membrane permeability assay (PAMPA), is one order of magnitude higher than that of Rh123 [16], and RhB has been shown to equilibrate faster with lipid multilamellar vesicles than Rh123, pointing to faster transmembrane movement [12]. For a series of rhodamine dyes, including RhB and Rh123, faster transmembrane movement correlated with higher stimulation of P-gp ATPase activity in reconstituted proteolipossomes [12]. This is in line with the inverse correlation observed between air–water partition coefficients (themselves highly correlated with the corresponding lipid–water partition coefficients) and the Michaelis–Menten constant for P-gp ATPase activation in a series of highly diverse compounds [17].

The present work seeks to bring insights into this matter, using atomistic molecular dynamics (MD) simulations of Rh123 and RhB interacting with simple membrane models. MD simulations of fluorescent membrane probes have been useful to obtain better understanding regarding their behavior when inserted in lipid bilayers, with unique detail [18,19,20]. Of particular relevance is the transverse location and orientation of the dyes in the bilayer, which will control the access to P-gp binding pocket and thus influence the binding site with which the molecule interacts. In this study, we address Rh123 and RhB in the presence of 1-palmitoyl-2-oleoyl-*sn*-glycero-3-phosphocholine (POPC; Figure 1f) bilayers. For validation of the simulations, we also measured the POPC/water partition coefficients of the two probes, which can be compared with estimates from free energy profiles calculated from the simulations.

Both probes possess ionizable groups, implying that they can exist in different ionization states, depending on pH. In acidic media, both nitrogen atoms of Rh123 are doubly protonated, resulting in an overall charge of +1 (structure in Figure 1)). This form may lose one of the amine protons, leading to the neutral species depicted in Figure 1a, with a reported p*K*_a_ value of 7.2 [21]. For RhB, the equilibrium constant between the cationic form with protonated carboxylic group (Figure 1e) and the zwitterion with negatively charged carboxylate (Figure 1d) has been estimated in aqueous solution from absorption measurements as *K*_a_ = 6 × 10^−4^ (p*K*_a_ = 3.2) at 25 °C [22]. More recently, p*K*_a_ values of 3.1 [23], 3.7 [24] and 4.2 [25] have been reported. Above this pH value, RhB exists in an overall neutral form, with an equilibrium between the zwitterionic form and the corresponding lactone (Figure 1c). The zwitterion is predominant in more polar media (such as water and short-chained alcohols), whereas lactone becomes more abundant in solvents with decreased formation of solvent-dye hydrogen bonds, and therefore less stabilization of the zwitterion (such as in *n*-octanol and in ramified alcohols) [26]. Because the conjugation of the chromophore is interrupted in the lactone, this is a colorless, non-fluorescent compound [1,27]. For both rhodamines, it should be noted that the abovementioned p*K*_a_ values concern aqueous solutions, and may not reflect entirely the distribution of bilayer-inserted dyes. While some groups have developed sophisticated procedures to carry out constant pH simulations [28,29], these schemes have limited practicability and complicated integration with the most common MD simulation packages, and therefore are currently not established for general use. For this reason, the conventional approach is still the independent simulation of the different ionization states, and therefore five species (two forms of Rh123 and three of RhB) are separately addressed here.

## 2. Results

As described in the detail in Section 3, three types of MD simulations were carried out. Firstly, systems composed of a single Rh solute were simulated in a box of ~1200 water molecules, solely for the purpose of topology testing and validation (see Section 3.2). The results of this preliminary study are not shown here, apart from the validated topology and final structures, which are available as Supplemental Material.

Subsequently, 4 Rh dyes of each type were added to a previously equilibrated bilayer containing 128 POPC lipids and 5140 water molecules, as described in Section 3.2. For each Rh species, three independent systems were thus prepared and simulated for 200 ns (Rh 123) or 500 ns (Rh B), without restraints (see Section 3.3). The results described in Section 2.1, Section 2.2 and Section 2.3 below were obtained from analysis of the final 100 ns (Rh 123) or 300 ns (Rh B) of these simulations.

Finally, for each studied Rh species, a set of 41 simulations with harmonic restraint of solute position at different locations across the bilayer normal was prepared. From the five sets (one for each Rh species) of 41 simulations, free energy profiles were calculated (see Section 3.4 for details). The use of the last 100 ns of each 120-ns run for sampling provided adequate profile convergence. These data are presented and discussed in Section 2.4, in comparison with experimental partition data.

A first glimpse of the interaction between the rhodamine dyes and POPC bilayers comes from observation of simulation snapshots, such as the final configurations depicted in Figures S1 and S2, supplementary materials. All solute molecules show clear interaction with the bilayer. While some of them adsorb to the interface, most display a location near (or just slightly more internal than) the lipid headgroups. A significant number manage to insert more deeply, especially for the neutral forms. A quantitative assessment of the behavior of probe molecules throughout the simulations is presented in the following subsections.

### 2.1. Location and Orientation of Rhodamine Dyes

**Figure 2:**
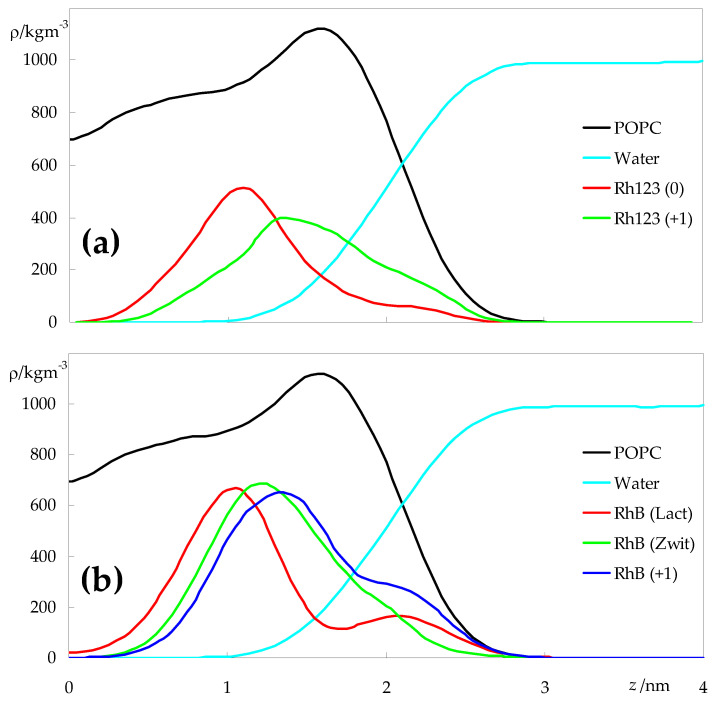
Symmetrized mass density profiles across the direction normal to the bilayer plane (*z*) of the different species, for Rh123- (**a**) or RhB-containing systems (**b**). *z* = 0 corresponds to the center of the bilayer (center of mass of POPC molecules). Rhodamine profiles were multiplied by a factor of 16 to facilitate visualization.

Figure 2 shows the density profiles obtained for water, POPC and for the two rhodamine probes in all forms, across the direction normal to the membrane plane (*z*). From these profiles, there is a more or less wide distribution of the solutes in the different ionization states, comprising both regions where the water density is significant (|*z*| > 1.5 nm) and regions where there is very little water penetration ((|*z*| < 1.0 nm). Minor peaks or shoulders, apparent in some profiles, denote molecules that did not insert the bilayer during the simulation and remained adsorbed to the interface region, as apparent in Figures S1 and S2. Overall, it can be confirmed that, as membrane probes, Rh123 and RhB report heterogeneity of molecular bilayer environments. For both dyes, the charged forms (Rh123 (+1) and RhB (+1)) are found in more external locations compared to their neutral counterparts. However, for RhB, the difference between the charged and overall neutral zwitterionic forms is very slight, whereas the lactone form is the one found on average closest to the bilayer center, even displaying non-negligible probability of location near *z* = 0.

**Figure 3:**
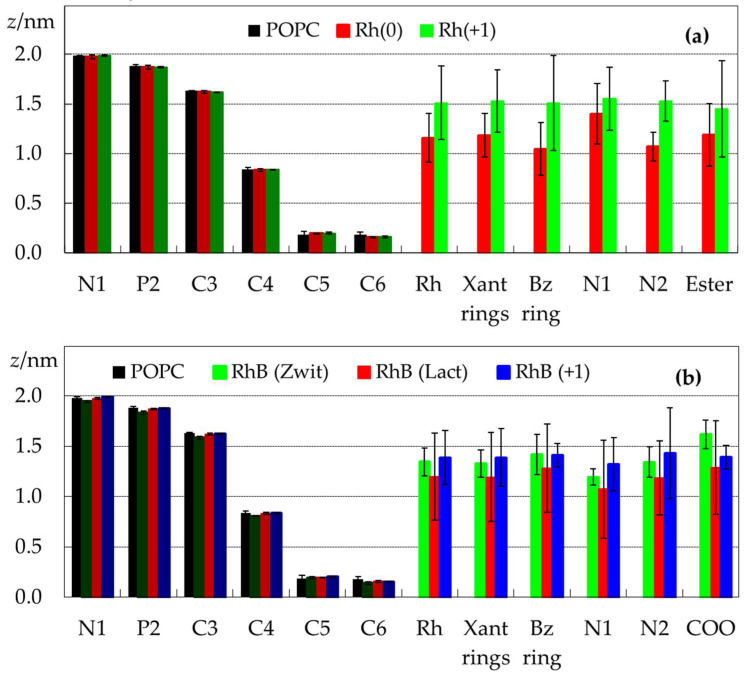
Average positions of selected POPC (columns labeled N1 to C6, darker colors) and rhodamine (columns labeled Rh to Ester/COO, lighter colors) atoms/atom groups, calculated for the simulations in the presence of Rh123 (**a**) or RhB (**b**). For atom numbering, please refer to Figure 1. For polyatomic groups, the average position of the center of mass is shown (Rh refers to the whole rhodamine molecule). Positions of POPC atoms in the absence of dye are also shown in both panels in black, for the sake of comparison.

This outermost position of the ionized and zwitterionic forms is also visible in the plots of Figure 3, which depict transverse positions relative to the center of the bilayer for selected POPC and rhodamine atoms and atom groups. For Rh123, the average positions of the center of mass for the ionic and neutral forms are (1.5 ± 0.4) nm and (1.2 ± 0.3) nm, respectively. The corresponding values for the three RhB forms vary between (1.2 ± 0.4) for the lactone and (1.4 ± 0.3) nm for the cation. The relatively large dispersion in these values is related to the wide dye density distributions shown in Figure 2, as a wide range of *z* values are probed by the different molecules. For example, most RhB(lactone) molecules reside, on average, in the *z* ∈ [0.8 nm, 1.5 nm] range. However, one molecule was located very close to the centre, whereas two molecules aggregated and remained adsorbed to the membrane surface, without inserting (not shown). Although these behaviors of individual molecules are reflected in the results (visibly in the density profiles of Figure 2 and the error bars in Figure 3), by simulating a relatively large number of molecules across three replicates, it is expected that the calculated average properties will not be critically affected. 

Figure 3 also shows the average transverse positions calculated for selected atoms of POPC and Rh. The atomic positions of POPC do not undergo significant changes, with the possible exception of the headgroup atoms in the presence of RhB (Zwitterion). Indeed, for this form the average position of the P atom (often used to define the bilayer thickness) is (1.83 ± 0.02) nm, down from (1.88 ± 0.01) nm for pure POPC.

Clear differences can be observed between the corresponding atoms of the different forms of the dyes. All the atoms in Rh123 (+1) have a very close location, indicating that the plane of the xanthene rings has an average orientation approximately parallel to that of the bilayer plane. In this ionization state, only the ester group presents, on average, an orientation slightly turned towards the bilayer core, with an incipient internalization of the methyl group, visible in some of the configurations illustrated in the snapshots of Figure S1. For Rh123 (0), there are some differences, since the xanthene rings are approximately 0.2 nm above the benzoate ring. Additionally, within the xanthene rings, the outermost atom is the nitrogen which is bonded to a single hydrogen (N1).

Turning to RhB, similarly to Rh123, the xanthene and benzoic rings have similar transverse locations in all three forms. However, contrary to Rh123, on very close inspection, one can notice that the xanthene rings have slightly more internal average locations than the benzoic ring. Although the differences between the average positions of the two ring systems are smaller than the uncertainty associated with each of them, in all but one of the nine simulations this more interior location of the xanthene was observed (not shown), confirming that such conformations are typical of bilayer-inserted RhB. The effect is more pronounced for the lactone and zwitterionic forms (for which the difference in depth of the rings is ~0.9 Å) than for the cation (for which it is ~0.2 Å). Thus, RhB (+1), similarly to Rh123 (+1) should have an almost horizontal disposition in the bilayer, whereas in RhB (Zwitterion) and RhB (Lactone) the benzoic ring is slightly oriented towards the water medium.

**Figure 4:**
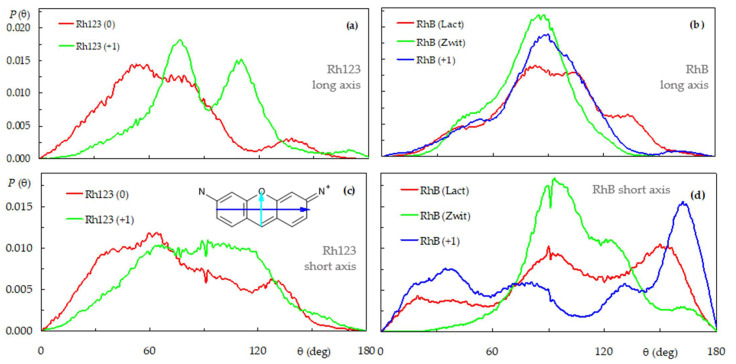
Distributions of the xanthene long- (**a**,**b**) and short-axis (**c**,**d**) tilt, relative to the bilayer normal for the Rh123 (**a**,**c**) and RhB (**b**,**d**) systems. See inset of panel (**c**) for long (blue arrow) and short (cyan arrow) axes definition.

These features may also be observed in the angular distributions of the tilts of two molecular axes of each rhodamine, the long and short axes of the xanthene (see definition in Figure 4, inset of panel (**c**)), relative to the bilayer normal. Broad distributions are recovered for all cases, pointing to multiple orientations that the dyes can adopt when interacting with the bilayer. As shown in Figure 4b, tilt angles close to θ = 90° are dominant for all forms of RhB (average values <θ> = 85.7°, 80.3° e 90.6° for cationic, zwitterionic and lactone forms, respectively), indicating orientations of this vector almost parallel to the bilayer plane. Conversely, consideration of all 12 Rh123 (+1) molecules produced a clearly bimodal distribution, with two peaks at θ ≈ 70° and θ ≈ 110° (Figure 4a). The two separated peaks indicate that this form of Rh123, differently from RhB, prefers to adopt an orientation where the long axis of the xanthene moiety is slightly tilted relative to the bilayer plane. The almost symmetrical distribution, with <θ> = 89.0°, is expected, because of the chemical equivalence of the N1 and N2 atoms and resulting symmetry of the xanthene group. In the neutral form of Rh123, the loss of one of the protons bound to N1 results in the breaking of the molecular symmetry. In turn, this leads to an asymmetrical distribution of the long axis tilt of Rh123 (0), with dominance of acute angles (<θ> = 67.3°), consistent with the more external location of N1 compared to N2 that was already visible in Figure 3a.

Regarding the short axis, Rh123 (+1) shows a very broad, symmetrical distribution, with <θ> = 86.9°, indicating that the collinear vector that links the centers of mass of the ring systems lies mostly parallel to the bilayer plane. In turn, this implies that the two ring systems have roughly identical similar average locations for Rh123 (+1), as mentioned above. Conversely, for Rh123 (0), acute short axis tilts dominate (<θ> = 73.5°), implying that the benzoate ring has a slightly more internal location than the xanthene, clearly visible in Figure 3a. On the other hand, the neutral forms of RhB have very broad, multimodal short axis tilt distributions, revealing different possible orientations. However, for all forms, obtuse average tilt angles are calculated (<θ> = 101.8°, 105.3° and 103.1° for the cation, zwitterion and lactone, respectively), consistent with slightly more external locations of the benzoic ring, compared to the corresponding xanthene moiety in each case.

### 2.2. Hydrogen Bonding

**Figure 5:**
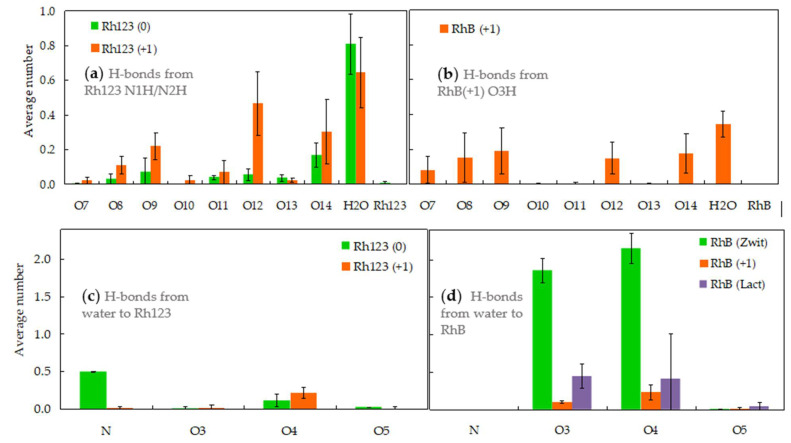
Hydrogen bonding involving rhodamine atoms as donors (**a**,**b**) to lipid atoms (numbering in Figure 1f), water or other Rh molecules, or as acceptors from water (**c**,**d**). In the latter case, the accepting Rh atom is identified (see Figure 1a–e for atom identification; “N” includes H bonds to N1 and N2). Panels (**a**,**c**) refer to Rh123, whereas panels (**b**,**d**) concern RhB. The data shown are average instant H bonds numbers per Rh donor or acceptor atom.

The subtle differences described in the previous section may be related to intermolecular interactions, such as hydrogen bonding with lipids and water, that in turn derive from the structural differences among the various forms of Rh123 and RhB. Rh123 contains −NH_2_ groups that can act mainly as donors, while the −NH group (in the neutral form) acts as a donor or acceptor of hydrogen bonds. Figure 5a shows that the Rh123 nitrogen atoms are involved almost continuously (summed average number of bonds per group close to or greater than 1) as hydrogen bond donors to the lipid, especially in its ionized form. For the neutral form, the average frequency of hydrogen bonding from −NH (N1) or −NH_2_ (N2) to the lipid O atoms is lower, in part due to the former having only one H atom for donation, and the latter having more internal locations, therefore more distant from the phosphate and glycerol groups of the POPC (Figure 3a). However, the frequency of bonds with water (either as a donor, or mainly as an acceptor) is maintained or even reinforced in the neutral form, which is related to the relatively external location of N1 (compared to N2), and its increased steric accessibility (being bonded to one, rather than to two hydrogens). 

Because the N atoms of RhB are attached to ethyl groups rather than H atoms, they cannot serve as hydrogen bond donors. The sole possibility of H bonding donation occurs through the carboxylic −OH group in Rh (+1). As shown in Figure 5b, this group has a similar pattern of H bonding to water and POPC phosphate/carbonyl/ester atoms as that of the −NH and −NH_2_ groups of Rh123, with predominance of the sterically less impeded POPC phosphate (O8, O9) and carbonyl (O12, O14) acceptor atoms. However, the overall number of H bonds is noticeably diminished, probably related to the more internal location of RhB (compared to Rh123 (+1)) and to having a sole H atom for donation. 

H bonding from water to RhB atoms varies considerably among its different forms. While RhB (+1) and RhB (lactone) act as H acceptors from water with moderate probability, RhB (zwitterion) carboxylate O atoms are permanently saturated, establishing on average close to the maximal 2 H bonds from water. This elevated frequency stems from the especially external location of these atoms (on average, more external than the corresponding atoms of any other form of either Rh123 or RhB) and also from their increased steric accessibility (both atoms being attached to a sole atom in the molecule). While the N atoms of Rh123 are mostly involved as H bond donors to water as discussed above, in the neutral form they may also act as acceptors from the solvent, Figure 5c. This interaction stems chiefly from the more accessible and external N1 atom (not shown). At variance, the N atoms of all forms of RhB show negligibly low H bond acceptance from water. This is probably caused mainly by their sterical hindrance, resulting from their attachment to two C_2_H_5_ groups, rather than a single H atom, as in N1 of Rh123(0).

### 2.3. Effects on Selected Lipid Properties

**Figure 6:**
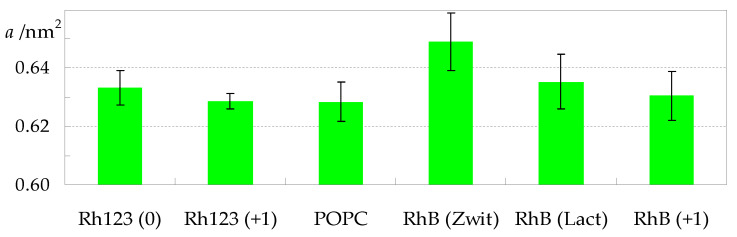
Average area per POPC for all studied systems.

As expected for all membrane probes, RhB and Rh123 affect host bilayer properties, with effects detectable at the 3.1 mol% concentration used in this study. Figure 6 illustrates the variation in average area in the bilayer plane per POPC molecule (a), across the different systems. While the value obtained in the absence of dyes (a = (0.627 ± 0.007) nm^2^) agrees with results from both experimental and simulation works [30,31], our main focus lies on the comparison among the different systems addressed here. As shown in Figure 6, although the value obtained for POPC is the lowest, the differences to other systems are not significant, with the exception of RhB (Zwitterion), for which a = (0.649 ± 0.010) nm^2^ was calculated.

**Figure 7:**
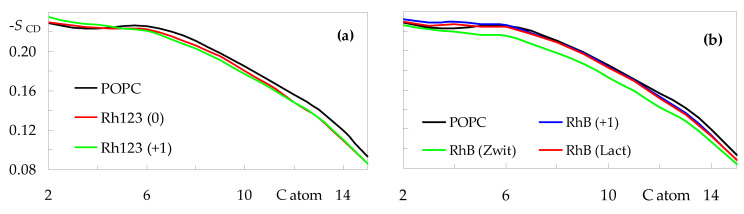
Order parameter profiles for the *sn*-1 acyl chain of POPC in the presence of rhodamine probes. (**a**): Rh123; (**b**): RhB. For the sake of comparison, the curve obtained for pure POPC is also shown in both panels.

To address more specifically how rhodamine dyes impact the hydrocarbon core, we turn to the acyl chain order parameters. Figure 7 shows the *sn*-1 deuterium order parameter profiles calculated for all systems. The profile obtained for pure POPC agrees with those reported in the literature, both from experiments and from simulations [30,32]. While the incorporation of Rh123 or RhB does not induce dramatic changes to the overall shape, some effects are apparent. In general terms, the studied rhodamine dyes reduce the order of the atoms closest to the center of the bilayer (for n ≥ 6, where n is the C chain atom index), whereas they tend (with the exception of RhB (Zwitterion)) to induce a slight ordering of the upper regions of the acyl chain (n ≤ 4). The rigid xanthene ring system preferably locates near the top end of the acyl chains, contributing to their alignment with the z direction, normal to the bilayer plane. On the other hand, probe insertion in this shallow region of the bilayer creates a local void underneath, which may be filled by the ends of the chains of neighboring POPC molecules, which, in the process, become less aligned with the z direction. This would explain the disordering observed in the lower acyl chain segments. The latter effect is especially pronounced for the zwitterionic form of RhB, for which the profile is on average ≈0.012 lower in this region, compared to pure POPC. This correlates with the increased POPC molecular area and decreased bilayer thickness observed for this system.

**Figure 8:**
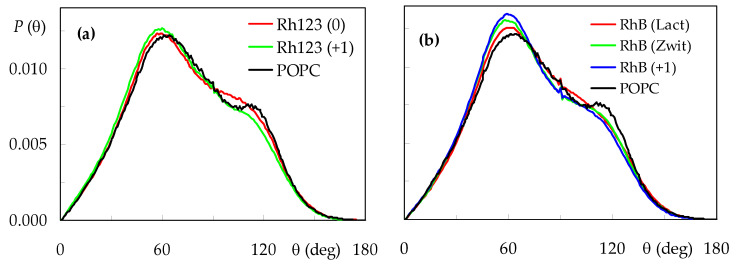
Distributions of the POPC P-N axis tilt relative to the bilayer normal, for the different systems. (**a**): Rh123; (**b**): RhB. For the sake of comparison, the curve obtained for pure POPC is also shown in both panels.

Finally, to investigate probe effects on the headgroup regions of the bilayer, we studied the orientation of the POPC P-N vector, θ. The tilt of molecular axis relative to the z direction has a typically broad distribution (Figure 8), which averages <θ> = 75.8° for pure POPC.

In the presence of rhodamine dyes, the distribution is slightly shifted to lower θ values, and a reduction in the shoulder at θ ≈ 110° is observed, together with an increase in the peak at θ ≈ 60°. This indicates that configurations where the choline N atom is located more internally than the phosphate P atom are less frequent when probe molecules are inserted, whereas those where the P-N vector protrudes ≈30° into the water medium are even more dominant. In molecular terms, the frequent location of rhodamine dyes near the headgroup region forces the P-N vector to be more aligned with the z direction. For the Rh123 probes, this effect is more visible for the cation (<θ> = 73.4°) than the neutral form (<θ> = 75.4°), whereas for RhB, the lowest average tilt is recorded for the cation (<θ> = 73.2°), followed by the zwitterion (<θ> = 74.1°) and lactone (<θ> = 75.3°). This means that, for each probe, the forms that have a more external location are the ones that induce a more significance perturbation of the POPC headgroup region.

### 2.4. Free Energy Profiles across the Bilayer and Relation to Experimental Partition

**Figure 9:**
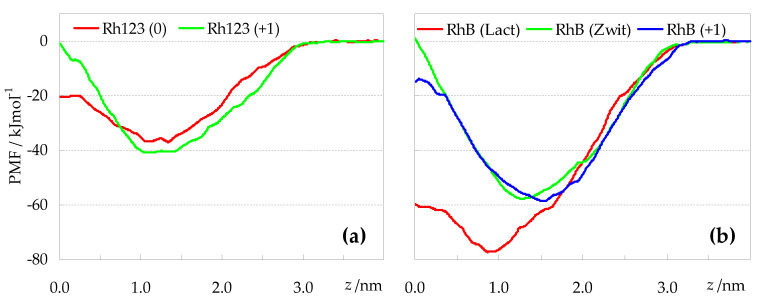
PMF profiles of rhodamine probes (obtained discarding the first 20 ns from analysis). (**a**): Rh123; (**b**): RhB.

Potential of mean force (PMF) profiles were obtained for all ionization states of both Rh123 and RhB (Figure 9a,b, respectively), using an umbrella sampling (US) procedure, as described in detail in Section 3.4. As discussed extensively in the literature, PMF profiles obtained from US simulations are susceptible to convergence problems [33,34,35,36,37]. To evaluate this possibility, we obtained PMF profiles using different regions of the 0 < *t* < 120 ns simulation time range, following the procedure outlined in Ref. [35], as described briefly in Section 3.4. Figures S3–S7 display the variation of the PMF profiles obtained discarding an initial (B panels) or final (C panels) time range, or using different 20-ns sampling intervals (D panels), for each rhodamine species. While the PMF curves began to converge as the time range used for analysis is extended in all cases, some variability is apparent. Particularly adequate convergence was observed for Rh123 (0) (Figure S3) and RhB (lactone) (Figure S5). In these cases, the PMF curves obtained using different 20-ns time ranges do not show a pronounced systematic behavior, leading to a small and/or non-monotonic variation of the resulting free energy barriers for translocation (equal to the difference in PMF values at *z* = 0 and at the energy minimum location) and desorption (equal to the difference in PMF values at *z* = 4 nm and at the energy minimum location), shown in the E panels of Figures S3–S7. In other cases, a tendency of the PMFs to evolve in a mostly monotonic manner can be detected. For example, Figure S6B,C show that, when the time range used for analysis of the RhB (zwitterion) is extended by discarding less time at the beginning or at the end (respectively) of the 120 ns simulation, the resulting PMFs show a downward or upward (respectively) tendency. In these cases, it could be argued that the profiles have not converged in 120 ns. However, we chose not to extend the sampling simulations, because even in the worst cases, the onset of convergence is apparent, and it is not clear whether a reasonable increase in the duration of the simulations would totally solve these issues. On the other hand, as shown in Figures S3–S7, in the initial part of the simulations (up to 20 ns) discrepant results are often obtained, probably because of lack of equilibration of the bilayer following pulling. Therefore, we chose to compare PMFs calculated discarding the initial 20 ns. The resulting profiles are displayed in Figure 9a,b for the Rh123 and RhB derivatives, respectively. In this comparison, we should consider the possibility of incomplete convergence and, indeed, bootstrapping error analysis indicates that most (*z*, PMF) points along the profile are subject to uncertainty of approximately ±20 kJmol^−1^ (Figures S3–S7, A panels). Still, the reasonable overlaps between *z* distributions in simulations with adjacent reference positions (Figure S8) do not reveal serious numerical problems in the calculation of the PMFs from these simulations, and we believe that some important conclusions can be drawn on relatively solid terms.

When comparing the profiles of Figure 9a (Rh123 species) with those of Figure 9b (RhB species), the most striking difference concerns the free energy minima. The latter are typically located at *z* ≈ 1.0 nm for RhB (lactone) and at larger *z* values, up to z ≈ 1.5 nm, for the other species, in very good agreement with the average Rh COM *z* locations of Figure 3. However, the depths of the free energy wells vary considerably from ~40 kJmol^−1^ for the Rh123 species, to ≈60 kJmol^−1^ for cationic and zwitterionic RhB, and almost 80 kJmol^−1^ for RhB (lactone). The latter species is confirmed as the most lipophilic of the studied probes, on account of its large stabilization deep in the hydrophobic core of the bilayer, and its translocation free energy barrier of <20 kJmol^−1^ is also the lowest (together with that of Rh123 (0)). This correlates with the density profiles of Figure 2, where Rh123 (0) and especially RhB (lactone) are the only derivatives showing non-negligible mass density near *z* = 0.

However, even excluding RhB (lactone) and comparing the other forms of RhB with those of Rh123, the deeper PMF profiles of the former point to higher lipophilicty. Thus, it is clear that ethyl substituents of RhB render it more hydrophobic than Rh123, despite the methyl esterification of the benzoate ring of the latter. Looking at the different ionization forms of each rhodamine (Rh123 (0) vs. Rh123 (+1); RhB (zwitterion) vs. RhB (+1)), differences in the PMF profiles are less marked, and manifest mainly in the magnitude of the translocation barrier, which is higher for the cationic Rh123 than for its corresponding neutral form. For RhB, the order of translocation barriers is reversed: the cationic form appears to have a lower activation for crossing to the opposite bilayer leaflet. This can be rationalized taking into account that the zwitterion, while having overall zero charge, presents relatively well separated charges in the xanthene rings (+1) and the carboxylate (−1). On the other hand, the cationic form, while maintaining the positive charge of the xanthene rings, has the carboxylate negative charge effectively neutralized. Therefore, in simple reductive terms, one may view the translocation of RhB (+1) as controlled by that of a single positive charge, whereas that of RhB (zwitterion) involves the transport of two separate charges (one positive, the other negative, the latter being highly localized), implying additional free energy cost. Indeed, the translocation barrier of zwitterionic RhB is clearly the highest among all studied probes.

One may try to relate these PMFs with experimental POPC/water partition coefficients, defined by

*K*_P_ = [Rh]*_l_*/[Rh]*_w_* (1)

where [Rh]*_l_* and [Rh]*_w_* represent the concentrations of probe in the lipid and water phases, respectively. A quantitative comparison with the simulation results is hampered because of the lack of a clear direct correspondence between the experimental *K*_P_ and the calculated Δ*G* values. The simple use of the thermodynamic relationship Δ*G* = −*RT*ln*K*_P_ ⇔ *K*_P_ = exp(−Δ*G*/*RT*) (*R* and *T* being the gas constant and absolute temperature, respectively) is probably not valid, because of different underlying reference states in the experimental and calculated *K*_P_, as well as arbitrariness in establishing the water/lipid interphase in the simulations. Still, in a number of computational works, *K*_P_ is taken as proportional to the integral of the exponential term along the reaction coordinate, thus accounting for both the depth and width of the free energy well [38,39,40,41,42]:

(2) $$K_{P}\propto \overset{a}{\underset{0}{\int }}\exp\left(-\frac{∆G\left(z\right)}{RT}\right)dz$$

In this equation, *a* represents a *z* value in a location within the water phase. We took *a* = 4.0 nm, but its choice is not critical, as for these regions, the exponential term (very close to 1) is much smaller than (and totally dominated by) the corresponding values for locations near the PMF minima.

**Figure 10:**
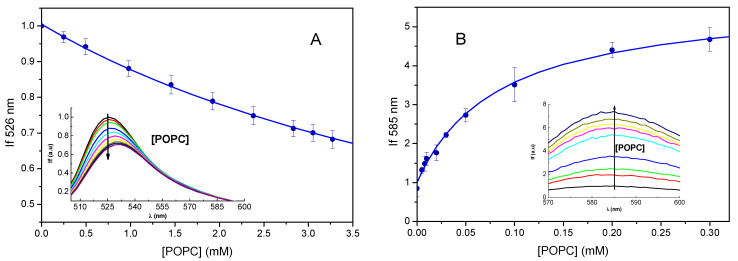
Variation in the fluorescence of Rh123 (**A**) and RhB (**B**) due to interaction with POPC LUVs. The fluorescence spectra are shown in the inset and the variation of the fluorescence intensity at the maximum wavelength when in the aqueous phase (526 nm for Rh123 and 583 nm for RhB) with the lipid concentration is shown in the main plot. The lines are the best fits of Equation (3), with *K*_P_ = 1.9 × 10^2^, and *S*_M_/*S*_W_ = 0.1 for Rh123, and *K*_P_ = 1.5 × 10^4^, and *S*_M_/*S*_W_ = 5.7 for RhB.

For experimental characterization of the partition of the rhodamines into POPC bilayers, we measured their fluorescence as a function of lipid concentration. The addition of POPC large unilamellar vesicles (LUVs) to Rh123 leads to a decrease in the fluorescence intensity (as previously observed [43]) and a red shift in the fluorescence spectra, Figure 10a, inset. The decrease in the fluorescence intensity at the maximum emission wavelength when in the aqueous media (526 nm) is represented in the main plot of Figure 10a, average and standard deviation of seven titrations. The variation with the lipid concentration was well described by a partition into the POPC bilayer, Equation (3), leading to *K*_P_ = 1.9 × 10^2^. The characteristic value of *K*_P_ was obtained from the average of ln *K*_P_ for the independent titrations [44], leading to *K*_P_ = 1.8 × 10^2^ with the confidence interval IC_90%_ [1.5 × 10^2^, 2.1 × 10^2^].

The association of RhB with POPC LUVs was also characterized, the results are shown in Figure 10b. In this case, the association with the POPC lipid bilayers leads to a very significant increase in the fluorescence intensity and a small red shift of the fluorescence spectra. The variation in the fluorescence intensity with the lipid concentration was used to calculate the partition coefficient of RhB (Figure 10b, main plot). The partition coefficient obtained from five independent experiments was *K*_P_ = 2.2 × 10^4^, with the confidence interval IC_90%_ [1.4 × 10^4^, 3.5 × 10^4^]. 

The values obtained for the partition coefficients of both rhodamines are in good qualitative agreement with those obtained previously from their accumulation in MLVs composed of phosphatidylcholine and phosphatidylglycerol [12], with RhB showing higher affinity for the membrane than Rh123. However, while the reported ratio of RhB/Rh123 was only 2 (with *K*_P_ ≅ 7 × 10^2^ for Rh123 and 1.2 × 10^3^ for RhB), in this work one obtains a ratio of over two orders of magnitude. This difference may be due to the presence of 20% negatively charged lipid in the previous work, which favors electrostatic interactions for the positively charged Rh123, and not for the RhB zwitterion. In fact, the presence of the negatively charged lipid seems to decrease the affinity of RhB to the lipid membrane by more than an order of magnitude. 

We can now calculate the integrals of Equation (2) for the different Rh species, and compare their normalized values (divided by the integral value of the dominant Rh123 form, Rh123(+1)) to a “normalized” experimental *K*_p_ (i.e., *K*_P_(Rh)/*K*_P_(Rh123)). This is shown in Table 1.

Of course, the experimental *K*_P_ values combine contributions from different possible ionization states, while the corresponding estimates from simulation pertain to specific forms of each probe. Nevertheless, a difference of roughly two orders of magnitude is observed both between the experimental *K*_p_ of the rhodamines, and the computed integrals of exp(−Δ*G*(*z*)/*RT*) for RhB (zwitterion and cation) and Rh123 (cation), pointing to a good semi-quantitative accordance. From our simulations, while the partition coefficient of the lactonic form of RhB is expected to be considerably higher, it must be noted that, because this species is not fluorescent, the experimental *K*_P_ value of RhB does not reflect its contribution. In fact, the observation that the fluorescence intensity increases significantly upon interaction with the lipid membrane suggests that this species is not stabilized to a large extent.

Figure 10 also illustrates that the fluorescence of Rh123 is quenched upon interaction with lipid bilayers, whereas the opposite is observed for RhB. Non-radiative deactivation of rhodamines has been proposed to occur through a mechanism similar to twisted internal charge transfer (TICT), in which an electron is transferred from the amino group to the xanthene Pi system, to form R_2_N^·+^ - Xanthene^·−^, and the R groups are rotated about 90° [45,46]. In such hypothesis, the presence of doubly alkyl-substituted N atoms favors this TICT state, by increasing the electron-donating ability of the N atom and enhancing the mean ground state twist angle for the amino groups [46]. This is the probable reason for the much lower fluorescence quantum yield of RhB in water, compared to Rh123 (0.36 and 0.89, respectively [46]). Possibly, the internalization of the N atoms of bilayer-inserted RhB, leading to slower rotation of R_2_N^−^ moieties, would decrease the extent of TICT state formation, increasing the fluorescence. Regarding the quenching of Rh123 emission upon interaction with POPC, our simulations do not offer a direct explanation. This effect is probably the result of alterations in the electronic structure of the excited state, possibly related to the H_2_N^−^ groups, upon bilayer insertion.

## 3. Materials and Methods

### 3.1. Quantum Chemical Calculations

Optimized geometries of Rhodamine-123 and Rhodamine-B in different forms were obtained by density functional theory (DFT) using the hybrid exchange–correlation functional B3LYP [47,48] together with the 6-31G(d,p) basis set. Frequency analysis subsequently performed confirmed each optimized geometry as an energy minimum by the absence of imaginary frequencies. Partial charges for the optimized rhodamines were calculated from a least-squares fit to the electrostatic potential obtained at the same theory level, according to the Kollman and Singh schemes [49,50]. All quantum chemical calculations were conducted with the GAMESS-US software package [51,52].

### 3.2. MD Parameters and System Set-Up

All MD simulations and analyses were carried out using GROMACS, versions 5 and later [53]. The GROMOS 54a7 force field was employed, with POPC parameters from Poger et al. [54,55]. The single point charge model of water was used [56]. Basic topologies of rhodamine dyes were built using the Automated Topology Builder [57,58]. Subsequently, rhodamine atom charges from this preliminary description were replaced with the values that resulted from the quantum chemical calculations described in the preceding section. For each rhodamine species, a box consisting of a single solute and ~1200 water molecules was built. In the case of the cationic species, a chloride ion was added to the water phase. The resulting systems were simulated for 10 ns (under *NpT* conditions at 298.15 K and 1 bar, with protocol as described in [59]) and the trajectories were analyzed for angles, proper and improper dihedral average values and distributions, to check their conformity with the input topologies and the structures obtained in the quantum chemical calculations. Slight adjustments in equilibrium values and force constants were carried out in a small number of parameters, leading to full agreement within a standard deviation in all cases. The resulting topologies, including atom charges obtained as described above and all other relevant molecular parameters, are included as supplemental material.

Using standard GROMACS tools, a fully hydrated POPC bilayer (128 POPC: 5140 H_2_O) was built and equilibrated (see Section 3.3). For each rhodamine, three replicate systems were obtained, by random insertion of 4 solute molecules near the interface region (replicate 1) or inside the bilayer leaflets (replicates 2 and 3). The required number of chloride ions was added to the water medium in each simulation involving charged rhodamines, to ensure neutrality.

### 3.3. Unrestrained Membrane Simulations

All systems were simulated under *NpT* conditions at 298.15 K and 1 bar. Equilibration/production run protocols and other simulation options were as described elsewhere [59]. The pure POPC system was simulated for 100 ns, of which the last 50 ns were used for calculation of reference area/lipid, atomic positions and deuterium order parameter profiles. Systems containing Rh123 or RhB were simulated for 200 ns (of which the first 100 ns were used for equilibration, and the remainder for analysis) or 500 ns (of which the first 200 ns were used for equilibration, and the remainder for analysis), respectively. The final structures of each of the triplicate simulations run for the different solute species are shown in Figure S1 (Rh123) and Figure S2 (RhB) in Supplementary Materials.

### 3.4. Umbrella Sampling Membrane Simulations and PMF Calculation

The free energy of the system (Δ*G*), as a function of the reaction coordinate (defined as the distance between the center of mass of the rhodamine molecule and the local center of mass of the POPC bilayer, i.e., calculated using only the POPC molecules whose locations in the bilayer plane were contained in a 1.1 nm radius cylinder centered on the solute), is derived from the Mean Force Potential (PMF), obtained from a series of US simulations [60]. Each of these runs corresponds to a location where the solute is harmonically restricted to reference distances from the center of the bilayer, with intervals of 0.1 nm, up to a maximum distance of 4 nm. This restriction allows the molecule to sample the configuration space for the calculation of forces in this interval, even in places where its permanence would be unlikely.

For this purpose, a rhodamine molecule, initially in the aqueous phase, is firstly pulled to the center of the bilayer (*z* = 0), with a rate of 0.0005 nm/ps and a force constant of 500 kJmol^−1^ nm^−2^. After this procedure, a second pulling run was performed, in which the molecule was gently pulled in the opposite direction, using the same speed and force constant of the previous step, starting from *z* = 0 and ending at *z* = 4.0 nm. From this simulation, 41 configurations were extracted in which the molecule was approximately in each of the transverse positions between *z* = 0 and *z* = 4.0 nm, spaced 0.1 nm apart. This protocol was chosen to obtain the initial configurations for the umbrella sampling runs, in order to avoid the slow convergence of the free energy profiles observed in similar systems, when the final pulling is done from the water medium to the membrane interior [35]. For the five rhodamine dye forms, each of these 41 systems was simulated for 120 ns (totaling ~25 µs), using the same conditions as in the unrestrained runs, but imposing a harmonic restraint potential, centered in the reference position, with a force constant of 3000 kJmol^−1^ nm^−2^. The resulting simulations were checked for convergence (as described in the main text, see Figures S3–S8, supplementary materials) and analyzed using the Weighted Histogram Analysis Method [61,62] to produce the PMF profiles.

### 3.5. Materials

The lipid 1-Palmitoyl-2-oleoyl-sn-glycero-3-phosphocholine (POPC), was acquired to Avanti Polar Lipids, Inc. (Alabaster, AL, USA), Rh123 was from Acros Organics (Geel, Belgium) and RhB was from Riedel-de-Haën (Seelze, Germany). Purified water was used (first distilled and then passed through activated carbon filters and deionized by ion exchange cartridges in the equipment ARIOSO UP, from Human, Seoul, Republic of Korea), and additional solvents, salts and buffers were of analytical grade. 

### 3.6. Preparation of Large Unilamelar Vesicles (LUVs)

For the preparation of lipid unilamellar vesicles (LUVs), a solution of the desired lipid/probe mixture in chloroform/methanol (87/13, *v*/*v*), as described previously [63]. The solvent-free residue was hydrated at 40 *°*C with Tris-MES buffer (final concentration: 41.2 mM Tris-MES pH = 6.8, 50 mM KCl, 5 mM NaN_3_, 2 mM EGTA, 1.03 mM Ouabain, 2 mM DTT, and 10 mM MgCl_2_), previously heated. The size of the LUVs was analyzed by dynamic light scattering (Zetasizer Nano ZS, Malvern) being close to the filter pore size (100 nm diameter). All the LUV samples were stored at 4–8 *°*C and used within 1–2 weeks.

### 3.7. Association of Rh123 and RhB with Model Membranes

POPC LUVs were diluted to the chosen concentration with Tris-MES buffer and warmed to 37 °C. Rh123 and RhB were added from stocks in DMSO (final DMSO 1% *v*/*v*) by squirting the required volume into the LUV solution under gentle vortex. The final Rh123 concentration was 1 µM and that of RhB was 2.5 µM.

Fluorescence intensity was measured after 10 min incubation at 37 °C using a Cary Eclipse spectrofluorimeter (Varian) with a thermostatted multicell holder accessory or with a plate reader SpectraMax iD5 (Molecular Devices). Rh123 was excited at 495 nm and RhB was excited at 557 nm.

The fluorescence variation of Rh123 and RhB when associated with the membranes is well described by a simple partition equation:

(3) $$I_{f}=\frac{S_{w}+S_{M}K_{P}{\bar{V}}_{\mathrm{POPC}}\left[\mathrm{POPC}\right]}{1+K_{P}{\bar{V}}_{\mathrm{POPC}}\left[\mathrm{POPC}\right]}$$

where *S*_W_ and *S*_M_ are, respectively, the fluorescence property (fluorescence intensity at 526 nm or at 583 nm) when all fluorescent dye is in the aqueous phase and associated with the membranes, *K*_P_ is the partition coefficient between the aqueous and membrane phases, $\bar{V}$ is the molar volume of the lipid when in the membrane (0.8 dm^3^/mol) [64], and [POPC] is the lipid concentration.

## 4. Conclusions

In this study, we addressed the interactions of Rh123 and RhB with POPC bilayers, by both computational (MD simulation) and experimental (fluorescence spectroscopy) methods. Rh123 and RhB are structurally similar compounds, sharing a xanthenic ring system, attached to a benzoic group. Our results show that both molecules tend to insert in a relatively shallow location, slightly dependent on the ionization form. Both compounds orient with the xanthenic long axis parallel to the membrane plane. 

Differences arise when the relative location of the two ring systems are considered. In the cationic state, Rh123 shows identical transverse location of the two ring systems, while, for the corresponding neutral form, the benzoic ring is positioned deeper than the xanthene. For both forms of Rh123, the hydrophilic NH/NH_2_ groups are the most external parts of the molecule.

In contrast, the ethyl-substituted nitrogen groups of RhB cannot act as hydrogen bond donors and, for steric reasons, do not establish H bonds as acceptors from water, either. As a result, the N atoms have now more internal positions, and the most outward region of the zwitterionic and cationic forms of the probe is the carboxylic group in the benzoic ring. RhB also has a lactonic form, which has more internal locations in POPC bilayers, displaying non-negligible density near the membrane hydrophobic core and, crucially, a much reduced energy barrier for translocation. Compared to the species with open carboxyl group, this form is not expected to be dominant in the polar interfacial regions of the bilayer, in accordance with the observed fluorescence of the dye when interacting with membranes. However, it appears plausible that fast reversible isomerization between zwitterion and lactone may occur when the former species is transiently placed at a sufficiently deep location within the bilayer, where hydrogen bonding from water (which stabilizes the zwitterionic form) is virtually absent. In turn, this may foster its translocation to the opposite bilayer leaflet where, upon arriving at a shallower location, it might reconvert to the zwitterionic form. This could explain the reported higher permeability of RhB compared to Rh123, which has the carboxylate esterified with a methyl group, therefore intrinsically less polar than that of RhB, but, crucially, is unable to form a lactone.

The results obtained also contribute to elucidate the different behaviour observed for the two rhodamine derivatives as P-gp substrates, namely a more efficient transport of Rh123, while RhB shows a higher enhancement of P-gp ATPase activity at smaller substrate concentrations. The predicted increased permeability of RhB, discussed above, implies that P-gp cannot expel RhB out of cells fast enough, justifying it being the poorest cellular substrate of P-gp among similar rhodamine dyes. At the same time, the much increased partition of RhB to membranes leads to a significantly higher local concentration in the bilayer, explaining the higher apparent affinity for P-gp, as indicated by stimulation of P-gp ATPase activity.

## Figures and Tables

**Table 1:** “Normalized” (see text for details) POPC/water partition coefficients obtained from fluorescence spectroscopy experiments and MD simulation.

Experimental	Rh123		RhB		
Experimental *K*_P_	1.8 × 10^2 1^		2.2 × 10^4 2^		
“Normalized *K*_P_”	1		1.2 × 10^2^		
**Computational**	**neutral**	**cation**	**lactone**	**zwit.**	**cation**
Normalized integral ^3^	0.18	1.0	1.3 × 10^6^	6.2 × 10^2^	8.2 × 10^2^

^1^ IC_90%_: [1.5 × 10^2^, 2.1 × 10^2^]. ^2^ IC_90%_: [1.4 × 10^4^, 3.5 × 10^4^]. ^3^ Integrals calculated from PMFs of Figure 9, using Equation (2).

## Data Availability

Structures and topologies of the studied rhodamine compounds, for use with GROMOS96 54a7 force field, are available as supplemental materials (please see above).

## Supplementary Materials

The following are available online, Figures S1 and S2: final configurations of the unrestrained simulations; Figures S3–S7: PMF error and convergence analysis for the studied rhodamine species; Figure S8: histograms of the *z* coordinate for the umbrella sampling simulations used for the calculation of the PMF profiles; zip archive with coordinate and topology files for the studied rhodamines, for use with the GROMOS96 54a7 force field.

## References

[1] Beija M., Afonso C.A.M., Martinho J.M.G. (2009). Synthesis and applications of rhodamine derivatives as fluorescent probes. Chem. Soc. Rev..

[2] Lavis L.D. (2017). Teaching Old Dyes New Tricks: Biological Probes Built from Fluoresceins and Rhodamines. Annu. Rev. Biochem..

[3] Smith D., Artursson P., Avdeef A., Di L., Ecker G.F., Faller B., Houston J.B., Kansy M., Kerns E.H., Krämer S.D. (2014). Passive lipoidal diffusion and carrier-mediated cell uptake are both important mechanisms of membrane permeation in drug disposition. Mol. Pharm..

[4] Seelig A. (2020). P-Glycoprotein: One Mechanism, Many Tasks and the Consequences for Pharmacotherapy of Cancers. Front. Oncol..

[5] Sajid A., Lusvarghi S., Murakami M., Chufan E.E., Abel B., Gottesman M.M., Durell S.R., Ambudkar S.V. (2020). Reversing the direction of drug transport mediated by the human multidrug transporter P-glycoprotein. Proc. Natl. Acad. Sci..

[6] Lusvarghi S., Robey R.W., Gottesman M.M., Ambudkar S.V. (2020). Multidrug transporters: Recent insights from cryo-electron microscopy-derived atomic structures and animal models. F1000Research.

[7] Lee T.D., Lee O.W., Brimacombe K.R., Chen L., Guha R., Lusvarghi S., Tebase B.G., Klumpp-Thomas C., Robey R.W., Ambudkar S.V. (2019). A high-throughput screen of a library of therapeutics identifies cytotoxic substrates of P-glycoprotein. Mol. Pharmacol..

[8] Al-Shawi M.K., Polar M.K., Omote H., Figler R.A. (2003). Transition State Analysis of the Coupling of Drug Transport to ATP Hydrolysis by P-glycoprotein. J. Biol. Chem..

[9] Shapiro A.B., Ling V. (1997). Positively Cooperative Sites for Drug Transport by P-Glycoprotein with Distinct Drug Specificities. Eur. J. Biochem..

[10] Landwojtowicz E., Nervi P., Seelig A. (2002). Real-Time Monitoring of P-Glycoprotein Activation in Living Cells. Biochemistry.

[11] Chufan E.E., Kapoor K., Sim H.M., Singh S., Talele T.T., Durell S.R., Ambudkar S.A. (2013). Multiple Transport-Active Binding Sites Are Available for a Single Substrate on Human P-Glycoprotein (ABCB1). PLoS ONE.

[12] Eytan G.D., Regev R., Oren G., Hurwitz C.D., Assaraf Y.G. (1997). Efficiency of P-glycoprotein–Mediated Exclusion of Rhodamine Dyes from Multidrug-Resistant Cells is Determined by their Passive Transmembrane Movement Rate. Eur. J. Biochem..

[13] Emaus R.K., Grunwald R., Lemasters J.J. (1986). Rhodamine 123 as a probe of transmembrane potential in isolated rat-liver mitochondria: Spectral and metabolic properties. Biochim. Biophys. Acta Bioenerg..

[14] Baracca A., Sgarbi G., Solaini G., Lenaz G. (2003). Rhodamine 123 as a probe of mitochondrial membrane potential: Evaluation of proton flux through F0 during ATP synthesis. Biochim. Biophys. Acta Bioenerg..

[15] Scaduto R.C., Grotyohann L.W. (1999). Measurement of Mitochondrial Membrane Potential Using Fluorescent Rhodamine Derivatives. Biophys. J..

[16] Fischer H., Kansy M., Avdeef A., Senner F. (2007). Permeation of permanently positive charged molecules through artificial membranes—Influence of physico-chemical properties. Eur. J. Pharm. Sci..

[17] Seelig A., Landwojtowicz E. (2000). Structure–activity relationship of P-glycoprotein substrates and modifiers. Eur. J. Pharm. Sci..

[18] Loura L.M.S., Prates Ramalho J.P. (2009). Fluorescent membrane probes’ behavior in lipid bilayers: Insights from molecular dynamics simulations. Biophys. Rev..

[19] Loura L.M.S., Ramalho J.P.P. (2011). Recent developments in molecular dynamics simulations of fluorescent membrane probes. Molecules.

[20] Filipe H.A.L., Moreno M.J., Loura L.M.S. (2020). The Secret Lives of Fluorescent Membrane Probes as Revealed by Molecular Dynamics Simulations. Molecules.

[21] Duvvuri M., Gong Y., Chatterji D., Krise J.P. (2004). Weak base permeability characteristics influence the intracellular sequestration site in the multidrug-resistant human leukemic cell line HL-60. J. Biol. Chem..

[22] Ramette R.W., Sandell E.B. (1956). Rhodamine b equilibria. J. Am. Chem. Soc..

[23] Arbeloa I.L., Ojeda P.R. (1981). Molecular forms of rhodamine B. Chem. Phys. Lett..

[24] Wang P., Cheng M., Zhang Z. (2014). On different photodecomposition behaviors of rhodamine B on laponite and montmorillonite clay under visible light irradiation. J. Saudi Chem. Soc..

[25] Maurya N.S., Mittal A.K., Cornel P., Rother E. (2006). Biosorption of dyes using dead macro fungi: Effect of dye structure, ionic strength and pH. Bioresour. Technol..

[26] Hinckley D.A., Seybold P.G. (1988). A spectroscopic/thermodynamic study of the rhodamine B lactone ⇌ zwitterion equilibrium. Spectrochim. Acta Part A Mol. Spectrosc..

[27] Drexhage K.H. (1976). Fluorescence efficiency of laser dyes. J. Res. Natl. Bur. Stand. Sect. A Phys. Chem..

[28] Santos H.A.F., Vila-Viçosa D., Teixeira V.H., Baptista A.M., Machuqueiro M. (2015). Constant-pH MD Simulations of DMPA/DMPC Lipid Bilayers. J. Chem. Theory Comput..

[29] Radak B.K., Chipot C., Suh D., Jo S., Jiang W., Phillips J.C., Schulten K., Roux B. (2017). Constant-pH Molecular Dynamics Simulations for Large Biomolecular Systems. J. Chem. Theory Comput..

[30] Piggot T.J., Piñeiro Á., Khalid S. (2012). Molecular dynamics simulations of phosphatidylcholine membranes: A comparative force field study. J. Chem. Theory Comput..

[31] Poger D., Caron B., Mark A.E. (2016). Validating lipid force fields against experimental data: Progress, challenges and perspectives. Biochim. Biophys. Acta Biomembr..

[32] Vermeer L.S., De Groot B.L., Réat V., Milon A., Czaplicki J. (2007). Acyl chain order parameter profiles in phospholipid bilayers: Computation from molecular dynamics simulations and comparison with 2H NMR experiments. Eur. Biophys. J..

[33] Neale C., Bennett W.F.D., Tieleman D.P., Pomès R. (2011). Statistical Convergence of Equilibrium Properties in Simulations of Molecular Solutes Embedded in Lipid Bilayers. J. Chem. Theory Comput..

[34] Paloncýová M., Berka K., Otyepka M. (2012). Convergence of free energy profile of coumarin in lipid bilayer. J. Chem. Theory Comput..

[35] Filipe H.A.L., Moreno M.J., Róg T., Vattulainen I., Loura L.M.S. (2014). How to tackle the issues in free energy simulations of long amphiphiles interacting with lipid membranes: Convergence and local membrane deformations. J. Phys. Chem. B.

[36] Neale C., Pomès R. (2016). Sampling errors in free energy simulations of small molecules in lipid bilayers. Biochim. Biophys. Acta Biomembr..

[37] Lee B.L., Kuczera K. (2018). Simulating the free energy of passive membrane permeation for small molecules. Mol. Simul..

[38] MacCallum J.L., Tieleman D.P. (2005). Computer Simulation of the Distribution of Hexane in a Lipid Bilayer:  Spatially Resolved Free Energy, Entropy, and Enthalpy Profiles. J. Am. Chem. Soc..

[39] Paloncýová M., Devane R., Murch B., Berka K., Otyepka M. (2014). Amphiphilic drug-like molecules accumulate in a membrane below the head group region. J. Phys. Chem. B.

[40] Kiametis A.S., Stock L., Cirqueira L., Treptow W. (2018). Atomistic Model for Simulations of the Sedative Hypnotic Drug 2,2,2-Trichloroethanol. ACS Omega.

[41] Tang P.K., Chakraborty K., Hu W., Kang M., Loverde S.M. (2020). Interaction of Camptothecin with Model Cellular Membranes. J. Chem. Theory Comput..

[42] Piasentin N., Lian G., Cai Q. (2021). Evaluation of Constrained and Restrained Molecular Dynamics Simulation Methods for Predicting Skin Lipid Permeability. ACS Omega.

[43] Eytan G.D., Regev R., Oren G., Assaraf Y.G. (1996). The Role of Passive Transbilayer Drug Movement in Multidrug Resistance and Its Modulation. J. Biol. Chem..

[44] Paketurytė V., Petrauskas V., Zubrienė A., Abian O., Bastos M., Chen W.Y., Moreno M.J., Krainer G., Linkuvienė V., Sedivy A. (2021). Uncertainty in protein–Ligand binding constants: Asymmetric confidence intervals versus standard errors. Eur. Biophys. J..

[45] Vogel M., Rettig W., Sens R., Drexhage K.H. (1988). Structural relaxation of rhodamine dyes with different N-substitution patterns: A study of fluorescence decay times and quantum yields. Chem. Phys. Lett..

[46] Zhang X.F., Zhang Y., Liu L. (2014). Fluorescence lifetimes and quantum yields of ten rhodamine derivatives: Structural effect on emission mechanism in different solvents. J. Lumin..

[47] Perdew J.P. (1986). Density-functional approximation for the correlation energy of the inhomogeneous electron gas. Phys. Rev. B.

[48] Becke A.D. (1998). Density—functional thermochemistry. III. The role of exact exchange. J. Chem. Phys..

[49] Singh U.C., Kollman P.A. (1984). An approach to computing electrostatic charges for molecules. J. Comput. Chem..

[50] Besler B.H., Merz K.M., Kollman P.A. (1990). Atomic charges derived from semiempirical methods. J. Comput. Chem..

[51] Schmidt M.W., Baldridge K.K., Boatz J.A., Elbert S.T., Gordon M.S., Jensen J.H., Koseki S., Matsunaga N., Nguyen K.A., Su S. (1993). General atomic and molecular electronic structure system. J. Comput. Chem..

[52] Gordon M.S., Schmidt M.W., Dykstra C.E., Frenking G., Kim K.S., Scuseria G.E. (2005). Advances in Electronic Structure Theory: GAMESS a Decade Later. Theory and Applications of Computational Chemistry.

[53] Abraham M.J., Murtola T., Schulz R., Páll S., Smith J.C., Hess B., Lindah E. (2015). GROMACS: High performance molecular simulations through multi-level parallelism from laptops to supercomputers. SoftwareX.

[54] Poger D., Van Gunsteren W.F., Mark A.E. (2010). A new force field for simulating phosphatidylcholine bilayers. J. Comput. Chem..

[55] Poger D., Mark A.E. (2010). On the validation of molecular dynamics simulations of saturated and cis-monounsaturated phosphatidylcholine lipid bilayers: A comparison with experiment. J. Chem. Theory Comput..

[56] Berendsen H.J.C., Postma J.P.M., van Gunsteren W.F., Hermans J., Pullman B. (1981). Interaction Models for Water in Relation to Protein Hydration. Intermolecular Forces.

[57] Malde A.K., Zuo L., Breeze M., Stroet M., Poger D., Nair P.C., Oostenbrink C., Mark A.E. (2011). An Automated force field Topology Builder (ATB) and repository: Version 1.0. J. Chem. Theory Comput..

[58] Stroet M., Caron B., Visscher K.M., Geerke D.P., Malde A.K., Mark A.E. (2018). Automated Topology Builder Version 3.0: Prediction of Solvation Free Enthalpies in Water and Hexane. J. Chem. Theory Comput..

[59] Filipe H.A.L., Santos L.S., Prates Ramalho J.P., Moreno M.J., Loura L.M.S. (2015). Behaviour of NBD-head group labelled phosphatidylethanolamines in POPC bilayers: A molecular dynamics study. Phys. Chem. Chem. Phys..

[60] Torrie G.M., Valleau J.P. (1977). Nonphysical sampling distributions in Monte Carlo free-energy estimation: Umbrella sampling. J. Comput. Phys..

[61] Kumar S., Rosenberg J.M., Bouzida D., Swendsen R.H., Kollman P.A. (1992). The weighted histogram analysis method for free-energy calculations on biomolecules. I. The method. J. Comput. Chem..

[62] Hub J.S., De Groot B.L., Van Der Spoel D. (2010). g_wham—A Free Weighted Histogram Analysis Implementation Including Robust Error and Autocorrelation Estimates. J. Chem. Theory Comput..

[63] Samelo J., Mora M.J., Granero G.E., Moreno M.J. (2017). Partition of amphiphilic molecules to lipid bilayers by ITC: Low-affinity solutes. ACS Omega.

[64] Wiener M.C., White S.H. (1992). Structure of a fluid dioleoylphosphatidylcholine bilayer determined by joint refinement of X-ray and neutron diffraction data. III. Complete structure. Biophys. J..

