# Supplementary material for: Interactions between Rhodamine Dyes and Model Membrane Systems—Insights from Molecular Dynamics Simulations
Source: Molecules. 2022 Feb 19;27(4):1420. doi: 10.3390/molecules27041420 (PMC8876248; doi:10.3390/molecules27041420)
Supplement: Supplementary file 1 [file molecules-27-01420-s001.zip › Supplemental_Figures.pdf]

Interactions between rhodamine dyes and model membrane systems – insights from molecular dynamics simulations

Nisa Magalhães [1,2], Guilherme M. Simões [1,2], Cristiana Ramos [1,2], Jaime Samelo [1,2], Alexandre C. Oliveira [1,2], Hugo A. L. Filipe [1,3], João P. Prates Ramalho [4], Maria João Moreno [1,2,5] and Luís M. S. Loura [1,5,6,*]

[1] Coimbra Chemistry Center - Institute of Molecular Sciences (CQC-IMS), University of Coimbra, 3004-535 Coimbra, Portugal
[2] Department of Chemistry, Faculty of Sciences and Technology, University of Coimbra, 3004-535 Coimbra, Portugal
[3] CPIRN-IPG - Center of Potential and Innovation of Natural Resources, Polytechnic Institute of Guarda, 6300-559 Guarda, Portugal
[4] Hercules Laboratory, LAQV, REQUIMTE, and Department of Chemistry, School of Science and Technology, University of Évora, Rua Romão Ramalho 59, 7000-671 Évora, Portugal
[5] CNC—Center for Neuroscience and Cell Biology, and Department of Chemistry, Faculty of Sciences and Technology, University of Coimbra, 3004-535 Coimbra, Portugal
[6] Faculty of Pharmacy, University of Coimbra, 3000-548 Coimbra, Portugal
E-mail contacts: nisa-tamara@hotmail.com (NM), guisims8@gmail.com (GMS), cristianavramos95@gmail.com (CR), jsamelo@uc.pt (JS), ac_oliveira10@hotmail.com (ACO), hlfilipe@ipg.pt (HALF), jpcar@uevora.pt (JPPR), mmoreno@ci.uc.pt (MJM), lloura@ff.uc.pt (LMSL)
* Correspondence: lloura@ff.uc.pt

Supplemental Figures

Contents

Figure S1 - Final configurations of the unrestrained simulations containing Rh123 (0) and Rh123 (+1).

Figure S2 - Final configurations of the unrestrained simulations containing RhB (Zwitterion), RhB (Lactone), and RhB (+1).

Figure S3 - PMF error and convergence analysis for Rh123 (0).

Figure S4 - PMF error and convergence analysis for Rh123 (+1).

Figure S5 - PMF error and convergence analysis for RhB (Lactone).

Figure S6 - PMF error and convergence analysis for RhB (Zwitterion).

Figure S7 - PMF error and convergence analysis for RhB (+1).

Figure S8 - Histograms of the z coordinate for the 41 umbrella sampling simulations used for the calculation of the PMF profiles of the studied rhodamines across a POPC bilayer.

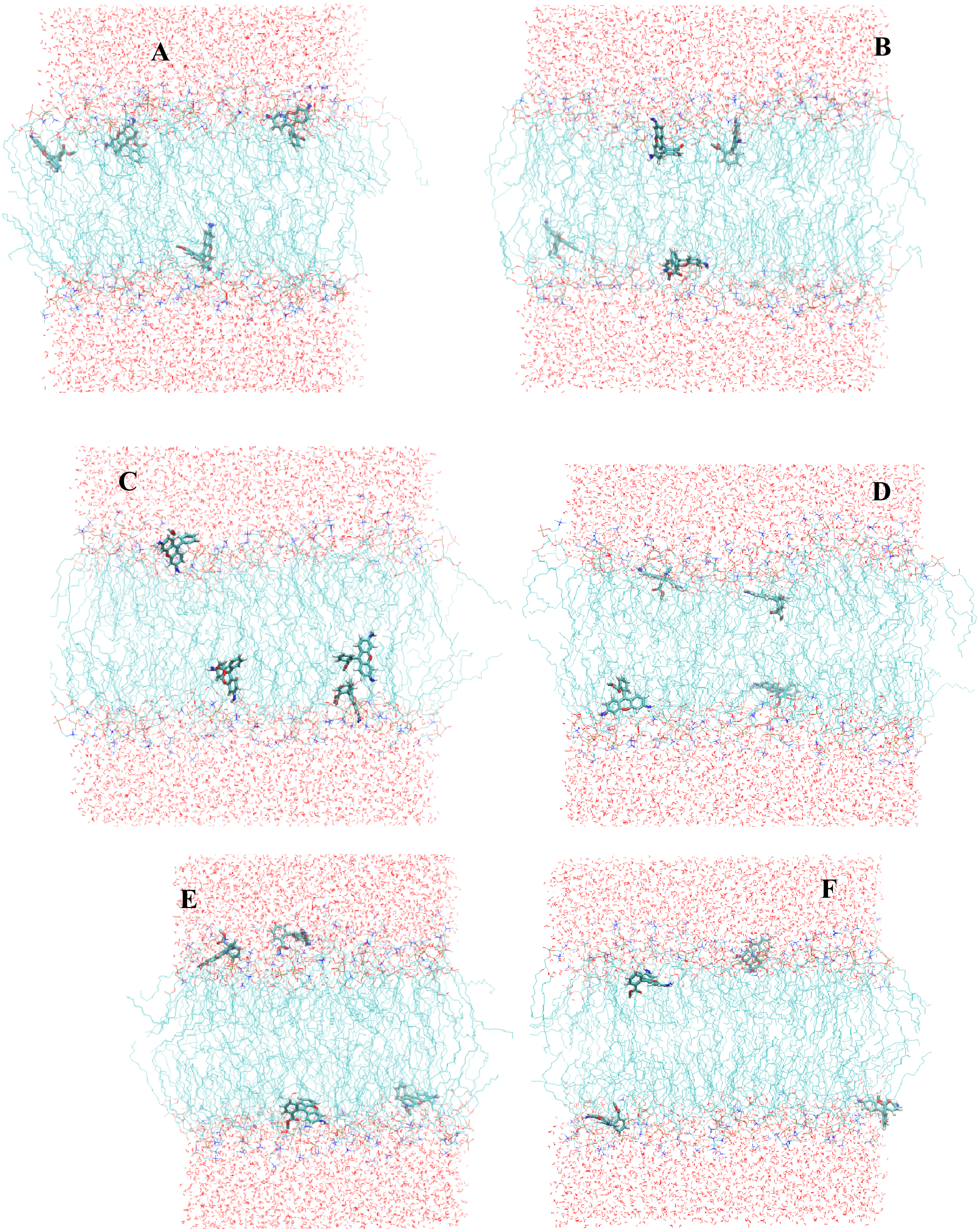

Figure S1 -Final configurations of the unrestrained simulations containing Rh123 (0) (A-C) or Rh123 (+1) (D-F), depicted with thicker lines. C, O, N, H and P atoms are shown in cyan, red, blue, white and tan colors, respectively. Note that the rectangular tetragonal boxes are not shown from the same side in all panels, for the sake of better visualization.

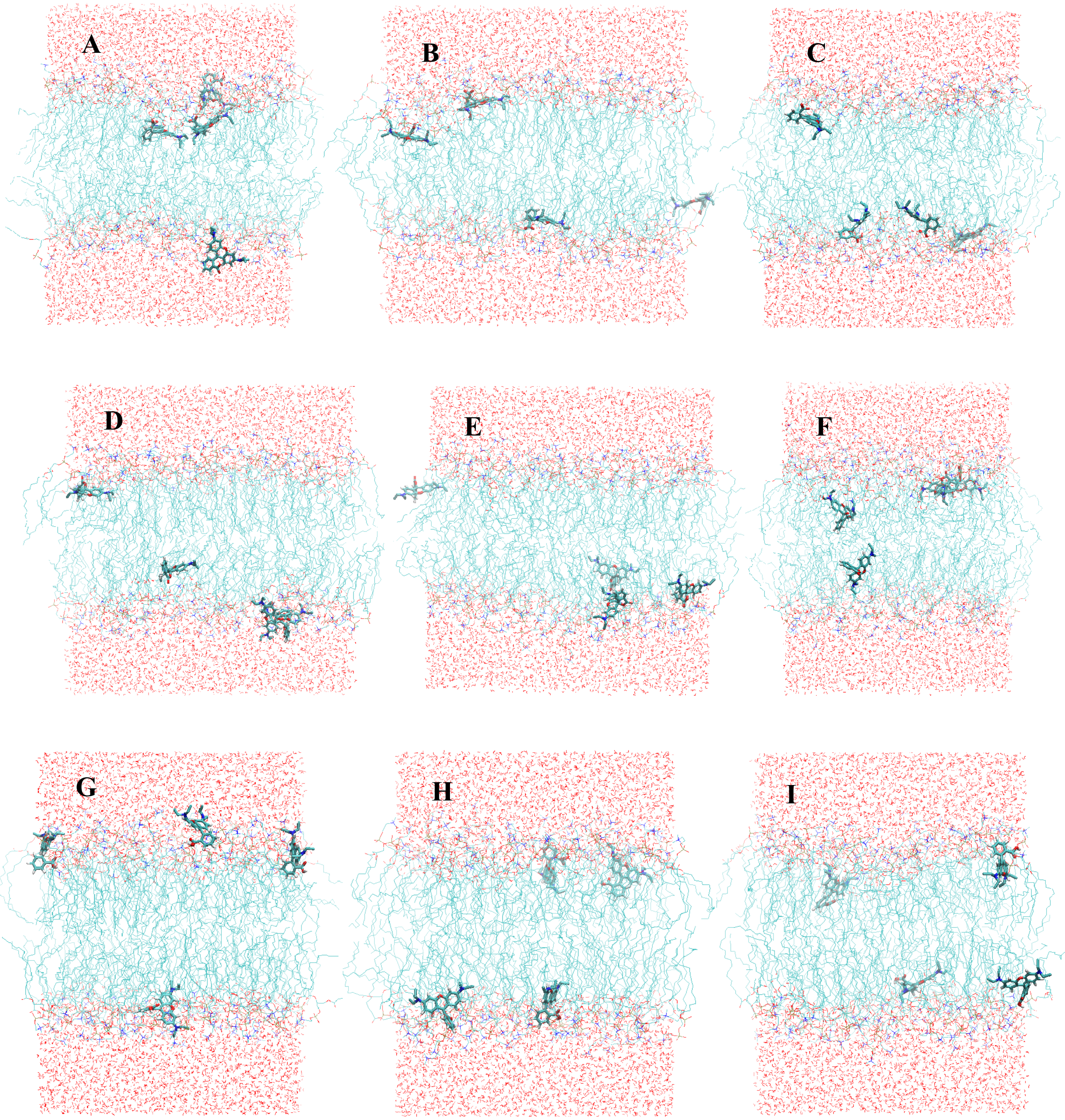

Figure S2 -Final configurations of the unrestrained simulations containing RhB (Zwitterion) (A-C), RhB (Lactone) (D-F), or RhB (+1) (G-I), depicted with thicker lines. C, O, N, H and P atoms are shown in cyan, red, blue, white and tan colors, respectively. Note that the rectangular tetragonal boxes are not shown from the same side in all panels, for the sake of better visualization.

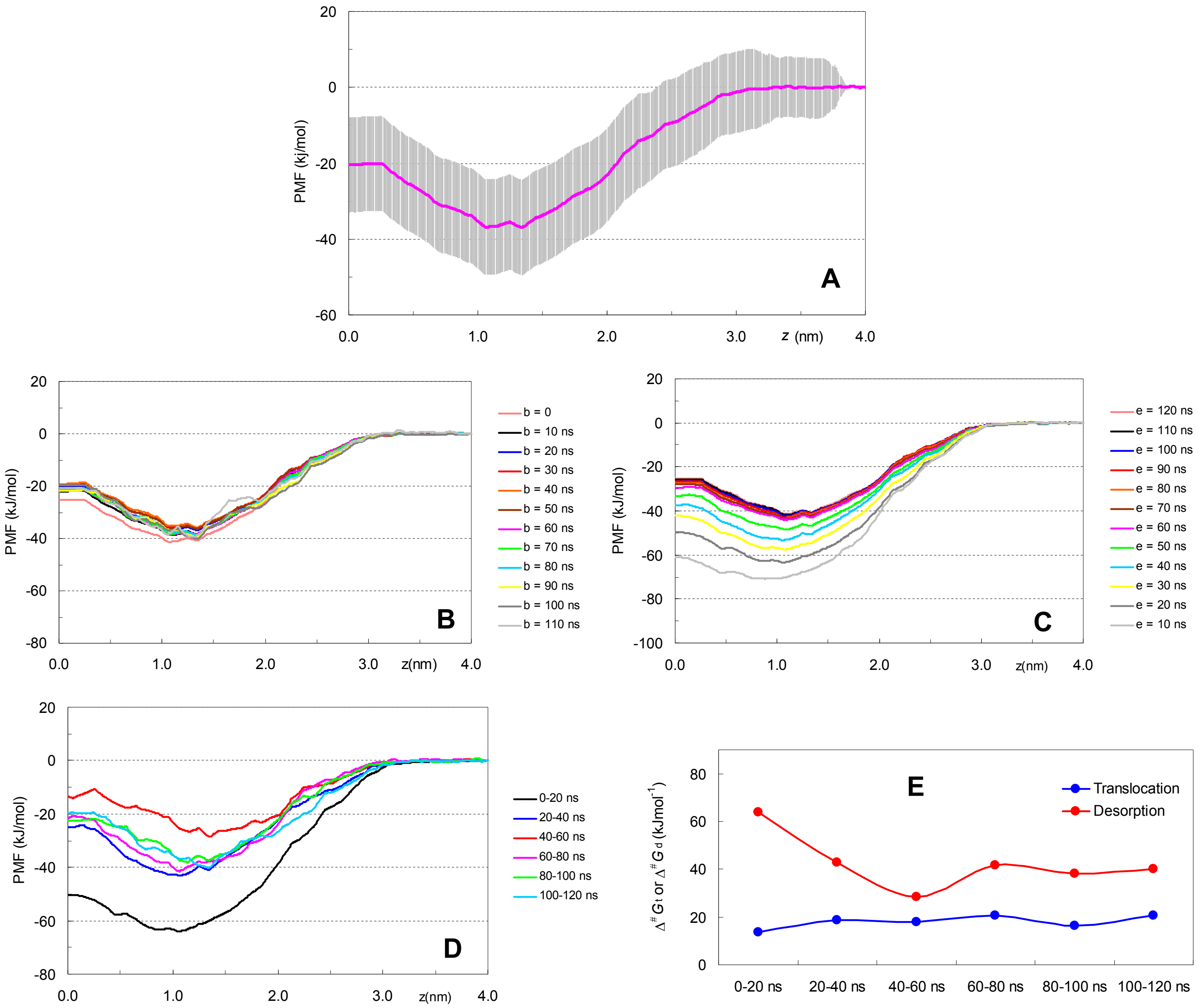

Figure S3 - PMF error and convergence analysis for Rh123 (0). (A): PMF curve calculated using the last 100 ns of the 120 ns sampling runs. (B): Convergence of the PMF profiles obtained discarding initial simulation times. b = *t* denotes that times before *t* were not considered for analysis. (C): Convergence of the PMF profiles obtained discarding final simulation times. e = *t* denotes that times after *t* were not considered for analysis. (D): PMFs obtained considering different 20 ns time intervals of the sampling simulations. (E): Translocation and Desorption free energies ($\Delta^{\#}G_t$ and $\Delta^{\#}G_d$, respectively) calculated from each of the PMF curves of panel D.

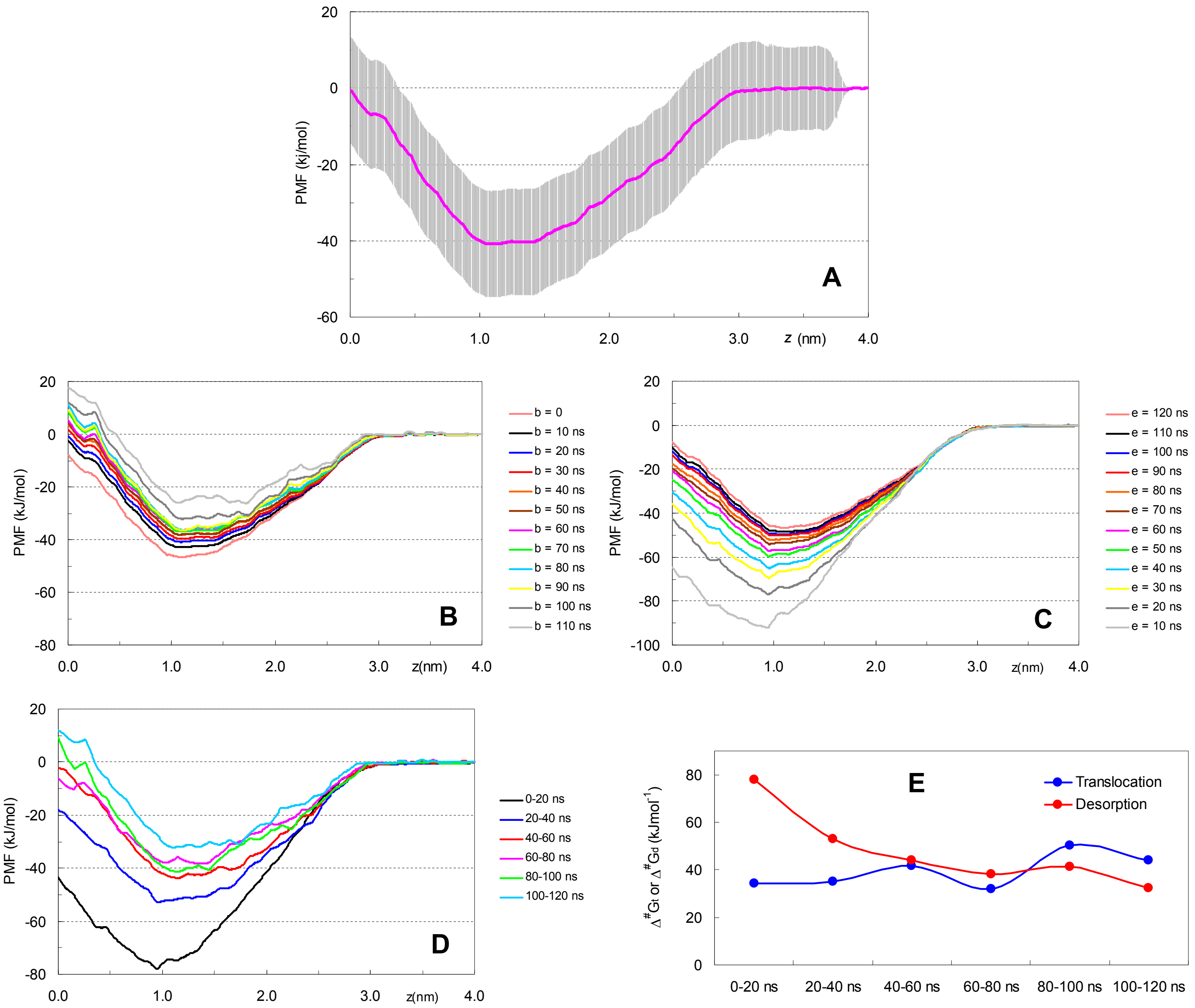

Figure S4 - PMF error and convergence analysis for Rh123 (+1). Panels A-E have the same meanings as in Fig. S3.

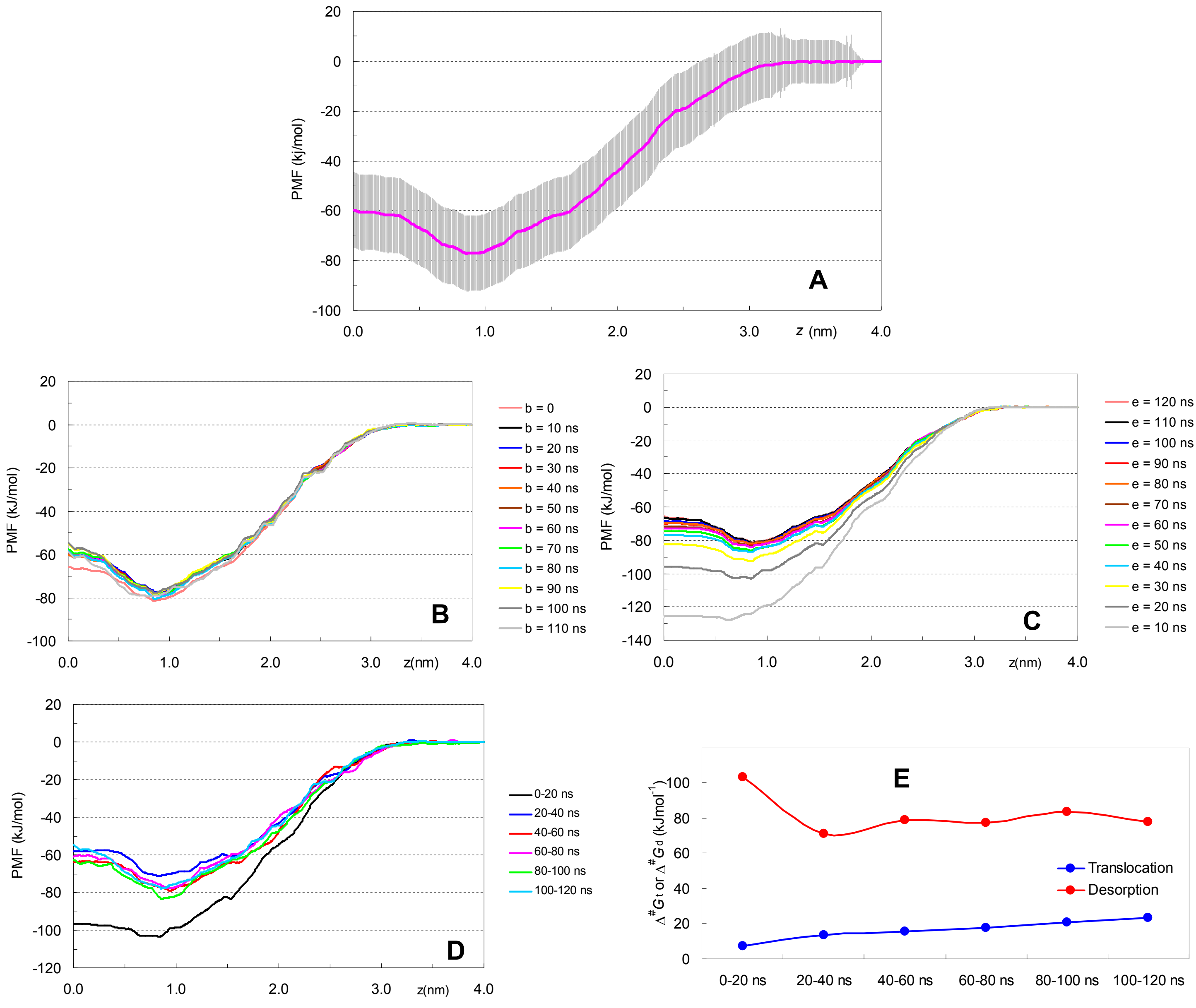

Figure S5 - PMF error and convergence analysis for RhB (Lactone). Panels A-E have the same meanings as in Fig. S3.

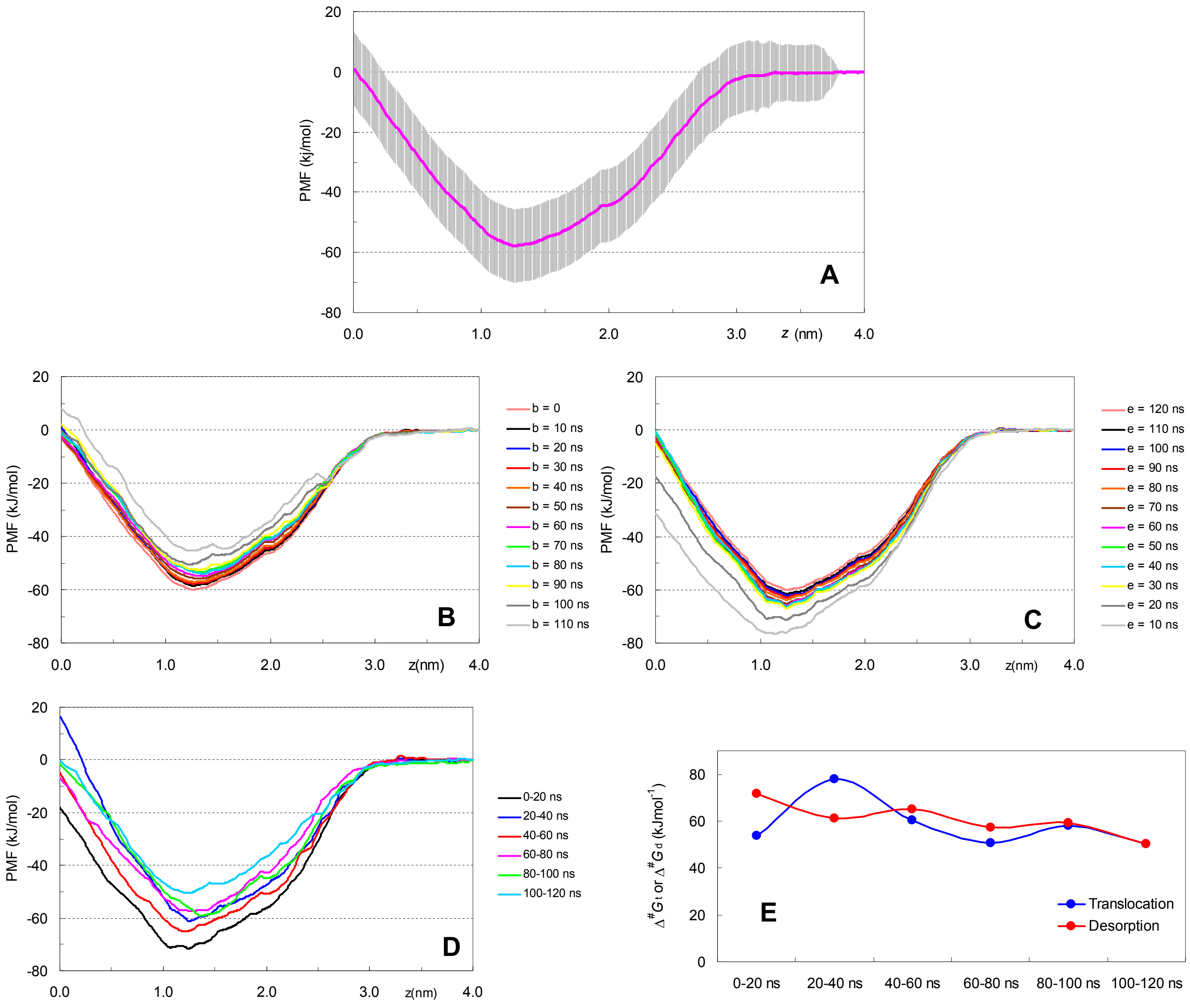

Figure S6 - PMF error and convergence analysis for RhB (Zwitterion). Panels A-E have the same meanings as in Fig. S3.

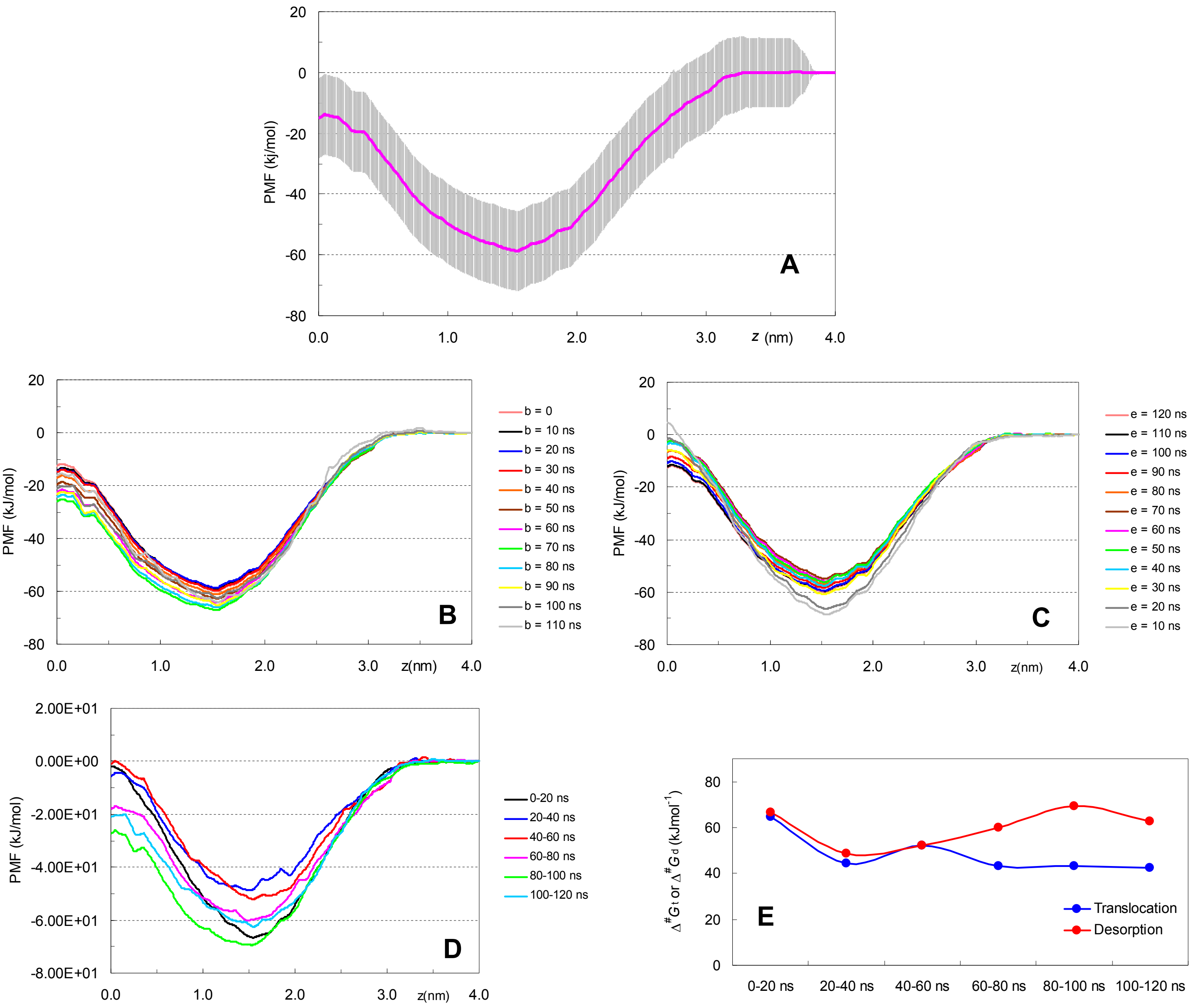

Figure S7 - PMF error and convergence analysis for RhB (+1). Panels A-E have the same meanings as in Fig. S3.

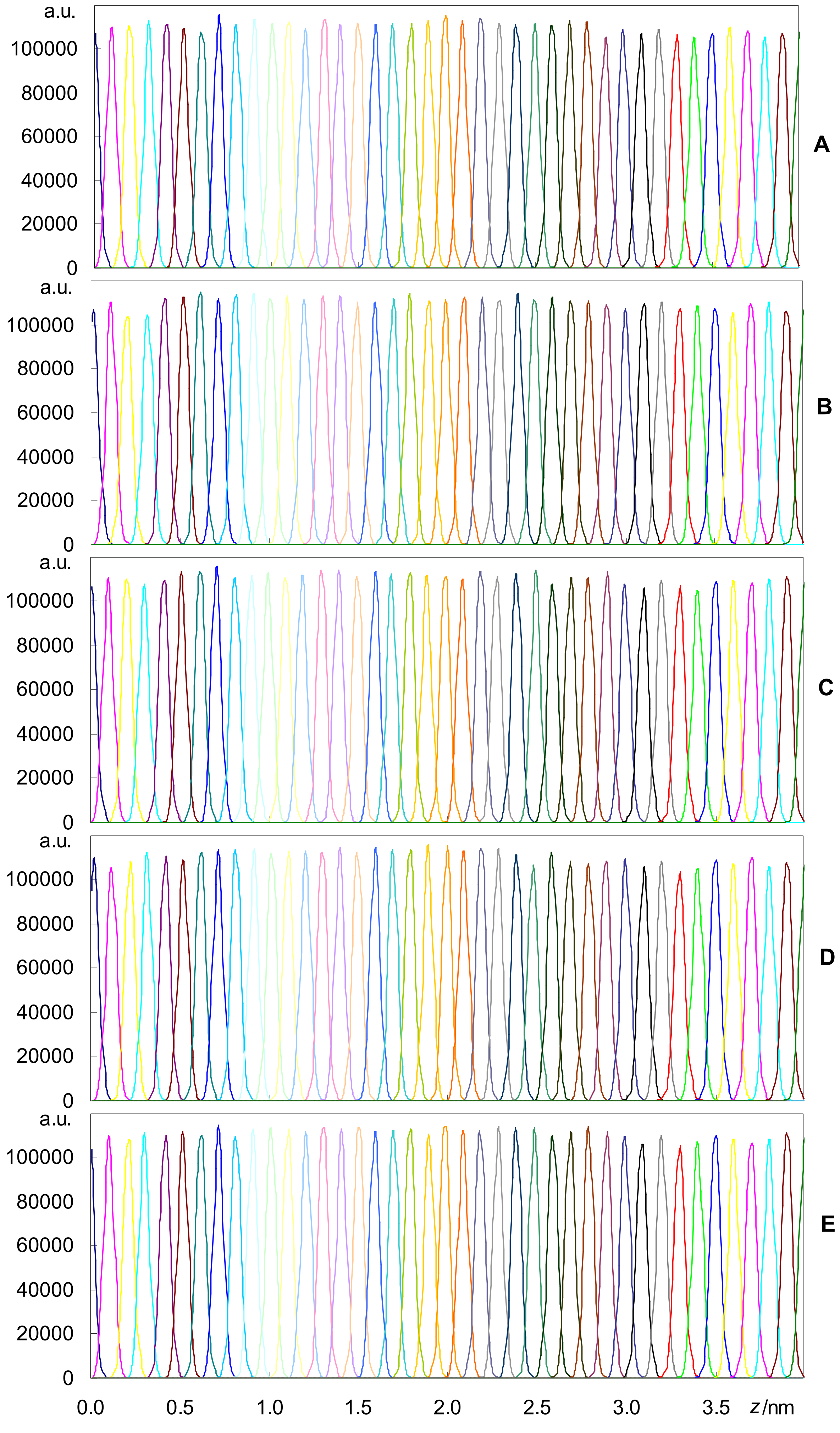

Figure S8 - Histograms of the z coordinate for the 41 umbrella sampling simulations used for the calculation of the PMF profiles of (A) Rh123 (0), (B) Rh123 (+1), (C) RhB (Lact), (D) RhB (Zwit), and (E) RhB (+1).
